# Pharmaceutical Residues in Sediments of a Coastal Lagoon in Northwest Mexico—Occurrence and Environmental Risk Assessment

**DOI:** 10.3390/jox14040093

**Published:** 2024-11-07

**Authors:** Oscar Fernando Becerra-Rueda, Griselda Margarita Rodríguez-Figueroa, Ana Judith Marmolejo-Rodríguez, Sergio Aguíñiga-García, Juan Carlos Durán-Álvarez

**Affiliations:** 1 Centro Interdisciplinario de Ciencias Marinas, Instituto Politécnico Nacional (CICIMAR-IPN), Av. IPN s/n Col. Playa Palo de Santa Rita, La Paz 23096, Mexico; fernando.becerra@oncotech.com.mx (O.F.B.-R.); gmrodriguez@ipn.mx (G.M.R.-F.); amarmole@ipn.mx (A.J.M.-R.); saguini@ipn.mx (S.A.-G.); 2 Departamento de Micro y Nanotecnologías, Instituto de Ciencias Aplicadas y Tecnología, Universidad Nacional Autónoma de México (ICAT-UNAM), Circuito Exterior S/N, Ciudad de México 04510, Mexico

**Keywords:** carbamazepine, ciprofloxacin, caffeine, La Paz lagoon, LC-MS/MS, inverse estuary, sulfamethoxazole, wastewater disposal

## Abstract

Contamination of marine ecosystems by pharmaceutically active compounds (PhACs) deserves more research since their environmental fate differs from that observed in freshwater systems. However, knowledge remains scarce, especially in semi-arid coastal regions of the Global South. This study investigates the occurrence and distribution of caffeine, carbamazepine, ciprofloxacin, and sulfamethoxazole in sediments from the La Paz lagoon, a coastal system in a semi-arid region of Mexico with inverse estuarine conditions. Samples of superficial sediments (0–5 cm depth) were collected from 18 sampling points distributed through the lagoon, encompassing sites heavily polluted by discharges of municipal sewage and 3 potentially pristine sites far from the urban and peri-urban zones. Also, a 25 cm length sediment core was taken and divided into 1 cm sub-samples to determine the deposition of target PhACs in the sediment bed through time. The extraction of the target PhACs was performed through the accelerated solvent extraction (ASE) technique and quantification was achieved using a validated HPLC-MS/MS analytical method. The concentration of caffeine, carbamazepine, ciprofloxacin, and sulfamethoxazole in superficial sediment oscillated in the range of 1 to 45 ng g^−1^ (dry weight). The highest mass fraction of target PhACs was detected in sites impacted by wastewater discharges. The caffeine-to-carbamazepine ratio was determined for the first time in marine sediments impacted by wastewater discharges, resulting in values from 4.2 to 9.12. Analysis of the 25 cm length sediment core revealed a high dispersion of caffeine, which was attributed to high water solubility, while antibiotics were predominantly detected in the upper 20 cm of the core. Risk quotients were calculated, observing low risk for caffeine, carbamazepine, and ciprofloxacin, while sulfamethoxazole presented high risk in all the sampling points. PhACs are retained in superficial sediments from a lagoon impacted by wastewater discharges, and the level of impact depends on the properties of the compounds and the TOC content in sediments. Risk assessments should be performed in the future considering the combination of pharmaceuticals and byproducts in marine sediments. This research emphasizes the importance of sewage management in preserving marine ecosystems in semi-arid regions in the Global South.

## 1. Introduction

Since the early 21st century, extensive research has been focused on understanding the occurrence and environmental fate of pharmaceutically active compounds (PhACs) in freshwater and terrestrial systems [1,2,3,4], along with their transport through food webs [5,6]. Monitoring studies in Western Europe and North America have led to the creation of Water Watch Lists for the early detection of pollutants in surface waters (Directive 2008/105/EC), encompassing pharmaceuticals (e.g., ciprofloxacin, diclofenac, and macrolides), organochloride pesticides, and sex hormones, due to their proven acute and chronic harmful effects on aquatic organisms [7,8], including mammals [9], at trace levels.

In contrast, in the Global South, particularly Africa [10] and Latin America [11], relatively few studies on the occurrence and toxicity of PhACs have been published. This scarcity hinders the development of appropriate regulatory frameworks that account for the unique characteristics and practices of such regions. For instance, wastewater reuse in agriculture and aquaculture [12,13,14], as well as the lack of proper wastewater treatment prior to disposal [15] are common challenges. Furthermore, little attention has been paid to the occurrence and distribution of PhACs in arid and semi-arid coastal zones, primarily located in developing countries in Africa and South America. Some research efforts have been made in the Middle East and Maghreb regions [16] but, to the best of our knowledge, there are no reports available for Latin America, particularly in arid regions such as Northern Chile and Northwestern Mexico.

In arid and semi-arid coastal areas, the xeric conditions foster the occurrence of inverse estuarine conditions during the warm season [17], leading to the depletion of dissolved oxygen in water and the formation of a halocline [18]. Under these temperature and salinity conditions, the uptake and effects of PhACs on marine species may be altered in unpredictable ways [19], emphasizing the urgent need to estimate the presence of PhACs in these coastal zones. Comprehensive monitoring studies comprising a wide range of PhACs are preferable but often not feasible, particularly in developing countries with limited access to sophisticated analytical techniques. Consequently, the use of refractory molecules, like carbamazepine, as markers of wastewater pollution in estuarine systems has been proposed [20]. Moreover, assessing the concentration of readily biodegradable molecules, like caffeine, in conjunction with recalcitrant pollutants (carbamazepine), can provide insights into wastewater depuration levels prior to disposal into estuaries [21,22].

In Mexico, drylands account for over three-quarters of the territory, with approximately 30% of the population living there under low-income conditions. Wastewater treatment is uncommon, and residues are often directly disposed of in freshwater reservoirs, the soil, or off the coast [23]. The city of La Paz, located in Baja California Sur, northern Mexico, serves as an illustrative case. There, sewage undergoes partial treatment in maturation ponds before being discharged into the estuarine zone, represented by the La Paz lagoon. It is well-known that this kind of treatment is unable to remove most PhACs from sewage [24], and after dilution in the lagoon, the pollutants can reach reach the bay through a tidal channel and eventually disperse into the Gulf of California Inverse estuarine conditions may restrict mixing and dilution processes, thereby favoring the accumulation of PhACs in suspended solids, sediments, and biota.

To the best of the authors’ knowledge, this is the first study reporting the presence of PhACs in marine sediments in northern Mexico and the first to perform this kind of analysis in arid coastal areas in Latin America. The occurrence of inverse estuary conditions in the La Paz lagoon, caused by the lack of freshwater inputs, results in limited attenuation of contaminants in the marine environment and poses significant risks to aquatic species. Therefore, this site may reflect an extreme case of environmental degradation, where marginally treated wastewater is received by the coastal environment, and the accumulation of pollutants in sediments is favored by the high salinity levels. For this study, caffeine, carbamazepine, ciprofloxacin, and sulfamethoxazole were selected, as the first two are markers of wastewater contamination and the extent of wastewater treatment, while the mentioned antibiotics are widely used by the Mexican population (an example of the most commonly used antibiotics in Mexico City can be found in [25]) as well as in aquaculture.

The aim of this work was to investigate the occurrence, distribution, and environmental risk of four specific PhACs in sediments from La Paz lagoon during the warm season using a validated analytical method based on liquid chromatography coupled to tandem mass spectrometry (HPLC-MS/MS).

## 2. Materials and Methods

### 2.1. The Study Area

**Figure 1:**
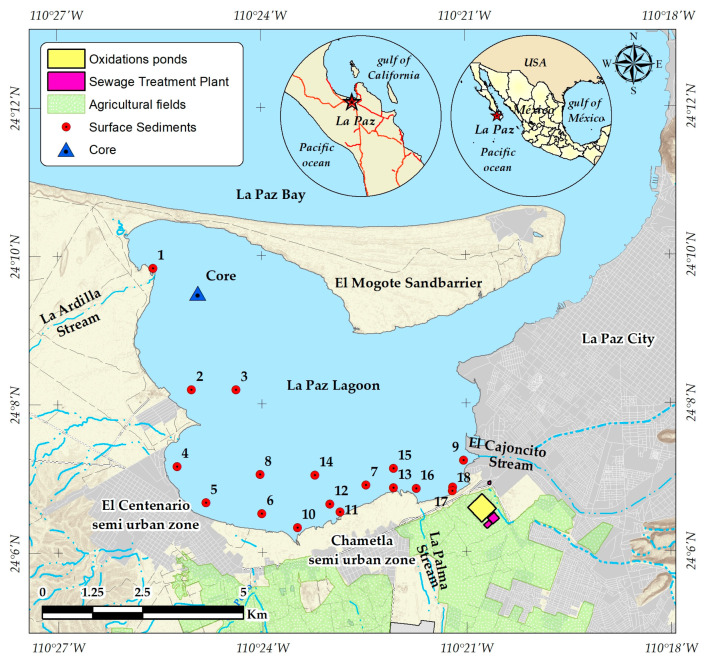
Location of the sampling points distributed through the La Paz lagoon. Map of La Paz Bay and La Paz Lagoon, Baja California Sur, Mexico. Shows sediment sampling points (red circles) and a sediment core (blue triangle), agricultural fields (green), oxidation ponds (pink), and sewage treatment plants (yellow).

The La Paz lagoon (Figure 1) is a shallow water body (45 km^2^), with depths ranging from 2 to 8 m) biogeochemically connected to the Gulf of California. It displays the typical morphological characteristics and processes of a semi-arid system, with an average annual rainfall of 200 mm. To the north, lies El Mogote, a sandy barrier that not only protects the lagoon from hurricanes and storms but also continuously supplies sandy sediments through wind and tides [26]. The lagoon is characterized by hypersaline waters and inverse estuarine circulation, with the shallowest part of the basin (averaging 2 m in depth) experiencing the highest evaporation rates and, consequently, the highest salinity levels [26]. There are two depressions, one in the north and the other in the south (approximately 7 and 4 m depth, respectively). Both are low-energy zones that facilitate the natural deposition of sediments, which remain undisturbed by natural or anthropogenic activities [27]. Surface water temperatures range from 20 °C in January to 31 °C in August, dropping to 21 °C, on average, from September to December. The mean annual salinity is 36.4 PSU, with higher values observed during the warm season (>38 PSU) and lower values in the fall (<35 PSU) [28].

Evaporation in the La Paz lagoon averages 215 mm per year, surpassing the maximum precipitation levels [29]. Chemically, the highest turbidity values are recorded during the fall and winter, increasing from the tidal channel toward the interior of the lagoon [28]. Peaks in chlorophyll-α levels typically occur twice a year—once in winter and again in summer—coinciding with phytoplankton blooms. The sedimentation rate in the northern depression of the lagoon was previously determined, using the radiometric method with ^210^Pb, as 6.5 mm per year [30]. The grain size and sediment distribution are divided into four zones: (1) the northern zone, with sandy sediments and a main deposition center enriched in silt and clay (sediment core site); (2) the southern zone, characterized by mud and a mixture of sands; (3) the central zone, with a sandy substrate and localized muddy areas presenting high organic matter content; and (4) the peripheral zone, where sediments are influenced by natural runoff and wastewater discharges [27]. In the peri-urban zone of La Paz city, comprising the semi-urban settlements El Centenario and Chametla, sewage is discharged into the La Paz lagoon throughout the year. Wastewater is either untreated or partially treated in maturation ponds before being released into the lagoon.

### 2.2. Chemicals

The US Pharmacopeia reference standards of caffeine, carbamazepine, ciprofloxacin, and sulfamethoxazole were brought from Sigma-Aldrich (St. Louis, MO, USA). Oasis HLB cartridges (200 mg solid phase, 6 cc) used for sample purification were purchased from Waters (Milford, MA, USA). The solvents used to extract, purify, and analyze the samples were HPLC-grade from Sigma-Aldrich. The physical and chemical properties of the target PhACs are displayed in Table S1.

### 2.3. Sampling of Sediments

Sampling points were distributed across the wastewater-impacted areas of the La Paz lagoon, along with three sites located far from the affected zones, considered pristine sites (LP 1 to LP 3 in Figure 1). All the samples were collected on the same day in the summer of 2016. For this, 50 mL polypropylene tubes were used to withdraw the sediment samples from the uppermost 5 cm layer. Prior to use, the tubes were thoroughly cleaned with 10% hydrochloric and nitric acid, followed by rinsing with deionized water, and then dried in a laminar flow hood. Additionally, a 25 cm sediment core was retrieved from the deepest zone of the lagoon, specifically from a depression at a depth of approximately 7 m. This core sample was obtained by free-diving and using a high-density polycarbonate tube with a diameter of 7.5 cm and a length of 30 cm. The polycarbonate tube, like all other sampling equipment, underwent a thorough cleaning process prior to use. To ensure the preservation of the samples, they were refrigerated during transportation to the laboratory and subsequently stored at 4 °C until preparation. The preparation process involved lyophilization at −80 °C for 48 h, followed by homogenization through milling in an agate mortar. The samples were then stored in PTFE vials at 4 °C until analysis.

At each sampling site, the water column was characterized using a multiparameter instrument to measure pH, salinity, and dissolved oxygen. In the laboratory, the sediment samples were analyzed for total organic carbon content and grain size distribution, as outlined in Table 1.

### 2.4. Analysis of Pharmaceuticals

#### 2.4.1. Extraction and Cleanup

The analytes were extracted from the sediments through the pressurized liquid extraction technique, using a ASE 150 device (Dionex, Sunnyvale, CA, USA). For each extraction, 1 g of dried sediment was mixed with 1 g of diatomaceous earth and transferred into 5 mL stainless steel cells. The samples were pre-heated at 70 °C for 5 min, and extraction was performed using methanol at 60 °C in three cycles of 5 min each. The flushing step was set at 60% of the extraction volume. The extracts (~20 mL) were then evaporated using ultra-high purity N_2_ to reduce the volume to approximately 3 mL before being reconstituted to 250 mL with HPLC-grade water.

Oasis HLB cartridges were preconditioned with 2 × 5 mL of methanol followed by 2 × 5 mL of water (pH = 2.5). The samples were loaded into the HLB cartridges without pH adjustment, using a vacuum at a flow rate of approximately 2 mL min^−1^. The cartridges were then washed with 5 mL of a 95:5 water mixture and dried under vacuum for 1 h. Elution was performed by applying 5 mL of methanol, allowing it to flow by gravity. The eluates were collected in amber glass tubes that had been pre-rinsed with methanol and then evaporated to dryness using ultra-high purity N_2_ at room temperature. Lastly, the samples were reconstituted to 1 mL with a 40:60 acetonitrile:0.1% formic acid mixture and transferred to amber glass chromatography vials for analysis.

#### 2.4.2. LC-MS/MS Analysis

Chromatographic separation was carried out using a high-performance liquid chromatography system 1200 Infinity series (Agilent Technologies, Santa Clara, CA, USA) equipped with a Zorbax SB-C18 column (4.6 × 150 mm, 5 µm particle size). The mobile phase consisted of 0.1% formic acid in water (A) and acetonitrile (B), with the following gradient: 60% A for the first 6 min, then decreased to 30% A over 0.5 min, and maintained from 6.5 to 12 min. Lastly, the composition was returned to 60% A, which was maintained until the end of the measurement at 18 min. The mobile phase flow rate was 0.4 mL min^−1^, and 5 µL of sample was injected for analysis. Electrospray ionization (ESI) was performed in the positive mode, with N_2_ as the drying gas at 300 °C and a flow rate of 11 L min^−1^. The nebulizer pressure was set to 50 psi, and the capillary voltage was 3000 V. Identification was achieved using multiple reaction monitoring (MRM) mode under the conditions specified in Table 2.

#### 2.4.3. Analytical Method Validation

The recovery of the method was evaluated by spiking the LP 1 sample with a mixture of the target PhACs at three concentration levels—30, 75, and 125 ng g⁻¹ (d.w.)—each in triplicate. A non-spiked sample was also analyzed to establish the background levels of the analytes. Matrix-matched calibration curves were elaborated to validate the analytical method within a working range of 10 to 500 ng mL⁻¹. The sediment sample LP 1 was used as the matrix for these calibration curves, with background analyte mass fractions subtracted accordingly. The limit of detection (LOD) was defined as three times the signal-to-noise ratio, while the limit of quantification (LOQ) was established as the lowest point within the linear range of the calibration curve. The results of the method validation, along with the calibration curves used for quantification, are presented in Table S2 and Figure S1, respectively.

### 2.5. Occurrence and Distribution of PhACs in Sediment Samples

The extent of pollution in surface sediments within the areas impacted by wastewater was determined by the analysis of sediment samples using the validated analytical method. As previously mentioned, the target PhACs were selected based on their utility as markers of estuarine pollution with wastewater: carbamazepine, being more recalcitrant, and caffeine, being more labile [31,32], while the antibiotics ciprofloxacin and sulfamethoxazole are highly used by Mexican population [25]. According to previous reports [33], the concentration ratio of these markers (*C.I._ratio_*), as defined in Equation (1)) was used to delineate the extent of wastewater treatment prior to its discharge into the estuarine system.

(1) $${C.I.\;}_{ratio}=\frac{\left[caffeine\right]\;}{\left[carbamazepine\right]}$$

The concentrations of the pharmaceutical residues are given in ng g^−1^.

Furthermore, the correlation between the mass fraction of the target PhACs in the sediment core samples and their usage by the local population was investigated, considering an average sedimentation rate of 6.5 mm per annum.

### 2.6. Risk Quotient Calculations

The potential risks posed by the target PhACs to exposed organisms were assessed by calculating the risk quotient (*RQ*) for marine species at various trophic levels. Following the guidelines of the European Commission [34], the *RQ* was determined by dividing the measured environmental concentration (*MEC*) by the predicted no-effect concentration (*PNEC*) for each compound (Equation (2)).

(2) $$RQ=\frac{MEC}{PNEC}$$

Here, the *RQ* values were calculated for all the concentrations measured in sediment samples, while *PNEC* values were retrieved from a review work focused on the chronic toxicity effects of PhACs on marine species [35]. Table S3 displays the factors considered for the determination of the *PNECs*, including endpoints and the assessment factor. Given the lack of toxicity studies in sediments, the *¡* values reported in aquatic environments (*PNEC_water_*) were further adapted to sediments (*PNEC_sediments_*) through Equation (3) [36].

(3) $${PNEC}_{sediments}=\frac{{PNEC}_{water}\times K_{d}}{\rho }\times 1000$$

where *K_d_* is the water–sediment distribution coefficient, taken from the literature (Table S4), and ρ is the density of the analyzed sediments (1260 kg m^−3^). PNECs were sought for different trophic levels and the lowest value for each target PhAC was used to determine the *RQ* values under the worst-case scenario.

## 3. Results and Discussion

### 3.1. Occurrence of Pharmaceutical Residues in Superficial Sediments

Of the four target PhACs, caffeine, ciprofloxacin, and sulfamethoxazole were detected in all the sampling points, while for carbamazepine, concentrations above the limit of quantification (1 ng g^−1^) were determined in 70% of the analyzed samples (Table 3).

Based on the analysis of the target PhACs, the contamination levels at the sampling sites were grouped as low, moderate, and high. The sampling points LP 1 to LP 3 were included in the first group. These sites were the farthest from the wastewater outflows, showing low total organic carbon (TOC) levels, which could limit the adsorption of organic molecules onto the sand particles predominant in the sediment bed (see Table 1). Subsequently, sites LP 4 to LP 8, and LP 10 to LP 16, showed contamination levels that were categorized as moderate. Conversely, high mass fraction levels of the target PhACs were quantified in sediment samples collected at points LP 9, LP 17, and LP 18, with LP 9 being located in the nearby of the water stream carrying sewage from La Paz city (Figure 1), while the sampling points LP 17 and LP 18 were near the outfall of the maturation ponds treating sewage prior to discharge into the coastal lagoon. Sediments from these sampling points presented the highest TOC concentration, which along with a high content of mud, would facilitate the adsorption of the organic pollutants on the sediment particles [37]. The high levels of the target PhACs in these sampling points coincide with the occurrence of heavy metals [27], attributable to the geochemical conditions in the zone.

The mass fraction of caffeine was in the range of 7.33 to 37.68 ng g^−1^ (d.w.) (Table 3). The highest levels found in this study coincide with those reported in marine sediments impacted by the discharge of sewage from densely populated areas. For example, maximum mass fraction levels of caffeine of 29.7 ng g^−1^ (d.w.) were reported in sediments from the San Francisco estuary [38], whereas in Todos Os Santos Bay and the north coast of Salvador, in Brazil, the highest caffeine mass fraction was found to be 23.4 ng g^−1^ (d.w.) [39]. Previous studies have reported the occurrence of caffeine in marine sediment within the range determined in this study [31,40]. However, it is worth noting that caffeine is not frequently detected in marine sediment samples due to its high solubility and low octanol–water distribution coefficient (Table S1), and because it is heavily degraded in sewage treatment plants prior to disposal off the coast [41]. Therefore, it can be found only in water–sediment systems receiving untreated or marginally treated sewage.

The antiepileptic carbamazepine was found in sediment samples within a mass fraction range of 1.13–1.86 ng g^−1^ (d.w.). Such levels are higher than those previously reported in marine sediments from mangrove [40] and other estuarine systems impacted by anthropic activities [42,43,44], where mass fraction levels varied from 1 ng g^−1^ (d.w.) in the rural region of Mahurangi, New Zealand, to 1.9 ng g^−1^ (d.w.) in sediments collected from the Augusta Bay, in Italy. As with caffeine, the levels of carbamazepine found in this study were similar to those reported in water–sediment systems directly impacted by marginally treated wastewater in the Todos Os Santos Bay and the North Coast of Salvador [39]. Furthermore, a recent study in the Odra River estuary [45], a highly industrialized area of northern Poland, reported mass fraction levels of carbamazepine in sediments higher than those obtained in this study (average 14.7 ng g^−1^, and maximum 79.8 ng g^−1^).

The mass fractions of caffeine and carbamazepine found in this survey reflect the low extent of sewage treatment in urban and peri-urban settlements surrounding the La Paz lagoon. Given that this is an inverse estuary system, the water column and sediments are characterized by high salinity (Table 1), fostering the partition of the organic molecules on the suspended solids and sediments [46]. Therefore, most of the organic pollutants in wastewater might be present in the sediment bed. Both carbamazepine and caffeine are considered to be markers of water pollution by municipal wastewater, with the former as a recalcitrant marker and the latter as a labile one [20,47]. According to previous studies in estuarine systems, the caffeine-to-carbamazepine ratio can indicate the extent of wastewater treatment prior to discharge [33]. Thereby, as a first attempt to establish the values for estuarine sediments impacted by marginally treated wastewater, we found that the caffeine-to-carbamazepine ratio in La Paz Lagoon ranged from 4.87 to 9.12 (Table 1). The highest values were found for sediments collected at sampling sites (LP 9, LP 16, LP 17, and LP 18) presenting the highest total organic carbon content (Table 1, 0.86–1.83%), namely areas heavily impacted by the sewage discharge. Although this kind of indicator is difficult to directly extrapolate from water to sediments, it can provide information for assessing the level of contamination in the sediment bed. Of course, further studies using a higher number of samples and analyzing different types of sediments are necessary to achieve more accurate values for the caffeine-to-carbamazepine ratio in marine and coastal sediments and thus more significant conclusions.

In the case of antibiotics, both ciprofloxacin and sulfamethoxazole were found at higher levels than carbamazepine and caffeine. The mass fraction of ciprofloxacin ranged from 4.09 to 31.04 ng g^−1^ (d.w.), which fell within the levels reported for estuarine environments impacted by reclaimed wastewater in the Gran Canaria Island [48], the Yangtze estuary [49], and the Odra River estuary [45]. Conversely, sulfamethoxazole presented the highest levels in all the sampling sites, going from 5.69 to 44.77 ng g^−1^ (d.w.). Previous studies have reported the prevalence of sulfamethoxazole over other antibiotics in estuarine sediments receiving wastewater discharges [50], displaying concentrations as high as 419 ng g^−1^ (d.w.). The high levels and spreading of antibiotics in sediments from the La Paz lagoon could be attributed to the wide use of these PhACs by the local population. In fact, both fluoroquinolones and sulfonamides are the most commonly used medications to treat enteric infections in Latin America [51]. Unfortunately, accurate information on the antibiotic usage patterns by the local population in La Paz city is not freely accessible, and thus, we were unable to establish reliable correlations between consumption and the presence of PhAC residues in the sedimentary environment.

Compared to ciprofloxacin, sulfamethoxazole can be retained on the organic domain of sediments at a higher extent, which is explained by its high K_ow_ value (Table S1), hence achieving the maximum levels in samples with higher content of organic carbon. The elevated salinity in the inverse estuarine system can also foster the partition of the organic molecules onto the sediments [46]. In the case of ciprofloxacin, retention in the sediments can occur through ionic interactions between the ionizable moieties within the molecule and the functional groups on the surface of the solid particles, which has been observed for this fluoroquinolone in clayey soils and other sediments [52,53]. The results obtained in this study are focused only on prescription pharmaceuticals and caffeine, while it opens the room to study the occurrence and behavior of over-the-counter PhACs (e.g., analgesics, contraceptives, and antihypertensives) as well as their metabolites in the sediment bed of this and other wastewater-impacted sites.

### 3.2. Distribution of PhACs in a Sediment Core

Analysis of the sediment core collected from the less impacted zone of the coastal lagoon showed the occurrence of caffeine and sulfamethoxazole throughout the core, displaying the highest mass fraction at the first 6 cm (Table 4). Conversely, carbamazepine was quantified in the first 15 cm, while the mass fraction of ciprofloxacin was below the limit of detection from the 24 cm depth. The mass fraction of caffeine in the upper part of the sediment core was slightly higher than that detected in superficial sediments from sampling points LP 1–3 in the pristine zone, which suggests the wide spread of this compound in the superficial layer of the sediment bed throughout the lagoon, even in sites far away from the pollution sources. For antibiotics, mass fraction levels found in the superficial layer of the sediment core (<9 cm depth) were higher than those observed in the sampling points of the pristine zone.

The sedimentation rate in the La Paz lagoon was previously determined as 6.5 mm per year [30], which means that the 25 cm core depth corresponds to approximately 46 years of sediment deposition. Ciprofloxacin was detectable to a depth of 21 cm, which coincides with its market authorization in the mid-1980s. Meanwhile, the use of sulfamethoxazole started in Mexico in the 1960s, and thus, it was detectable throughout the 25 cm depth, representing the site’s history from 1977/78. Caffeine was quantified through the sediment core due to its high water-solubility along with its extensive use for decades. Carbamazepine was released on the market in the 1960s [54], although its presence in the sediment core dates only to the early 1990s, suggesting that its distribution through the core might be impacted by other factors, such as the physical and chemical properties of the sediment.

The characterization of the sediment core displayed a higher mass fraction of total organic carbon at the surface, which could be controlling the retention of organic molecules [55]. The mass fraction of the target PhACs substantially decreased with depth (Table 4), being more evident for antibiotics and carbamazepine beyond a 20 cm depth, where the TOC mass fraction dropped below 0.05%. Such behavior can be explained by three factors acting alone or jointly: (a) the drop in the TOC mass fraction limits the retention of the organic molecules in deeper layers of the core; (b) the uptake of the antibiotics by benthic organisms, leading to the progressive disappearance of the molecules as the sediments bed grows; and (c) the mobilization of the superficial sediments by hydrodynamic forces [37]. The first two factors are the most plausible as the core was collected from a low-energy zone, where the sediment bed is hardly disturbed by natural or anthropic forces. Conversely, the degradation and uptake of ciprofloxacin and sulfamethoxazole by some benthic organisms have been previously reported in marine sediments [56,57], which would lead to a decrease in the antibiotic mass fraction levels in the lower parts of the core [58].

Caffeine presented a wide distribution through the sediment core, which provides evidence for its high mobility and is in line with that previously reported for freshwater sediments and the vadose zone of soil [32,55]. Considering the low partition of caffeine toward the solid phase, which is inferred by its low values of the log K_ow_ and log D parameters (Table S1), it is possible that this molecule is transported dissolved in water to deeper layers of the sediment core. This hypothesis could be more plausible over the translocation of the sediment particles through the core.

### 3.3. Environmental Risk Assessment

The risk quotient is an estimation of the probability of the occurrence of detrimental effects in organisms exposed to the target pollutants at the mass fraction levels quantified in the sediment samples. This parameter is determined by dividing the measured concentration of each target PhAC by the predicted no-effect concentration (*PNEC*) determined in chronic toxicity tests using different endpoints. The works reporting *PNECs* of the target pollutants in sediments are scarce, hence these values were inferred using the *PNEC* values corresponding to seawater and the water–sediment distribution coefficient (*K_d_*) of each compound (see Tables S3 and S4). Caffeine presented an extremely low risk for exposed benthic organisms (*RQ* < 0.01) in most of the sampling points, while the risk for carbamazepine and ciprofloxacin was determined as low, with *RQ* values between 0.01 and 0.1 (Table 5). In the case of sulfamethoxazole, risk quotient values (from 1.18 to 8.46) corresponded to a high environmental risk, which correlates with the high mass fraction of the antibiotic in all the sampling points.

The risk assessment obtained for target PhACs is consistent with that reported in sediments of the Odra River estuary and the Szczecin lagoon [45]. The presented environmental risk assessment is only an estimate, considering that the PNEC values used herein were determined for marine aquatic species. However, the target pharmaceuticals have been demonstrated to cause detrimental effects in benthic organisms at different trophic levels, using different endpoints. For example, mass fraction levels of carbamazepine in sediments over 50 ng g^−1^ have been correlated with mortality of polychaetes in long-term (14 d) laboratory studies [59]. This antiepileptic has been demonstrated to bioaccumulate and induce oxidative stress in *S. plana* and *D. neapolitana* at µg L^−1^ levels [60]. Also, the occurrence of carbamazepine in marine sediments, even at low levels (0.5–500 ng g^−1^), has been demonstrated to significantly increase the mitochondrial electron transport and the total lipid content while reducing the activity of the monoaminoxidase enzyme in *H. diversicolor* [59]. Moreover, recent studies have found higher bioaccumulation of carbamazepine in *M. galloprovincialis* during and up to 10 days after heatwaves, which resulted in oxidative damage and diminished immune response [61]. Antibiotics elicit important inhibitory effects on the microbiome of marine sediments at ng g^−1^ levels. For instance, ciprofloxacin can limit the capacity of the bacterial community to degrade polyaromatic hydrocarbons at levels as low as 0.4 ng g^−1^ [62]. Also, the concomitant occurrence of antibiotics and heavy metals in marine sediments, as happens in the La Paz lagoon [63], has been demonstrated to promote the expression of antimicrobial resistances in *Enterococci bacteria* [64], which is considerably hazardous in a water body systematically receiving poorly treated and raw wastewater.

## 4. Conclusions

This is the first study reporting the occurrence and distribution of pharmaceutical residues in marine sediments from a semi-arid area in the Global South. The occurrences of caffeine, ciprofloxacin, sulfamethoxazole, and carbamazepine in sediments indicate the environmental impact caused by the continuous discharge of marginally treated wastewater into the La Paz lagoon. The highest mass fraction levels of PhACs were found at sites near the coast, where municipal wastewater is discharged, although there was a uniform distribution of these contaminants across the coastal lagoon, indicating the translocation of contaminants most likely driven by the hydrodynamic conditions of the water body. The caffeine-to-carbamazepine ratio, traditionally used in aquatic estuarine systems, was calculated for the superficial sediments in the inverse estuary system of the La Paz lagoon, resulting in values ranging from 4.20 to 9.12. The values obtained in this work can be considered the first use of the caffeine-to-carbamazepine ratio in sediments impacted by raw wastewater, and further studies can corroborate these values and provide numbers for systems receiving treated wastewater.

The presence of antibiotics in the sediment core could be associated with their release in the market; although for carbamazepine this correlation was not so clear, hence it is possible that the physical and chemical properties of the sediment are playing a decisive role in its vertical distribution through the sediment bed. The mass fraction levels of caffeine, carbamazepine, and ciprofloxacin in sediments represented low to extremely low risk for benthic species, while for sulfamethoxazole, the risk was determined as high (RQ > 0.1). The risk that antibiotics pose to benthic organisms is certainly a matter of concern, and further monitoring studies must be carried out to quantify other PhACs to unveil the risk of toxic effects caused by mixtures of pharmaceuticals and their degradation byproducts. This study paves the way for understanding the occurrence and environmental fate of pharmaceutically active compounds in peculiar natural systems such as inverse estuaries.

## Figures and Tables

**Table 1:** Characterization of the sediments sampled in the La Paz (LP) lagoon.

Sample	pH	TDS (×10^3^ mg L^−1^)	Salinity (%)	D.O. (%)	TOC (%)	Sand (%)	Mud (%)
LP 1	8.4	28.3	37.8	98.3	0.13	95	5
LP 2	8.9	28.1	37.4	95.1	0.22	92	8
LP 3	8.2	28.5	38.0	96.1	0.09	93	7
LP 4	8.9	28.1	37.5	96.9	0.46	86	14
LP 5	9.2	28.1	37.5	98.9	0.58	87	13
LP 6	8.8	28.1	37.5	94.9	0.37	77	23
LP 7	8.2	28.3	37.6	91.4	0.33	95	5
LP 8	8.0	28.7	38.3	83.2	0.40	96	4
LP 9	8.4	28.4	37.9	87.0	0.86	83	17
LP 10	8.5	28.7	38.2	81.5	0.53	85	15
LP 11	8.6	28.6	38.1	84.6	0.77	83	17
LP 12	8.3	28.4	37.8	85.7	0.61	82	18
LP 13	8.8	28.5	38.0	95.8	0.84	81	19
LP 14	8.5	28.5	38.0	98.8	0.55	92	8
LP 15	8.4	28.8	38.5	87.9	0.43	95	5
LP 16	8.6	28.3	37.7	59.3	0.88	94	6
LP 17	8.2	28.4	37.8	54.8	1.23	85	15
LP 18	8.8	28.5	38.0	64.5	1.83	84	16

TDS: total dissolved solids, D.O.: dissolved oxygen, and TOC: total organic carbon.

**Table 2:** Optimum mass spectrometry parameters for the analytes.

Molecule	Precursor Ion (m/z)	Product Ion (m/z)	Fragmentation Voltage (V)	Collision Energy (eV)
Caffeine	195.1	138.1 110.1	100 80	16 18
Ciprofloxacin	332.2	314.2 288.2	135 110	17 20
Sulfamethoxazole	254	156 98	100 70	15 23
Carbamazepine	237.1	194.1 114.1	110 65	15 20

**Table 3:** Mass fraction levels of target PhACs in surface sediment samples from the La Paz lagoon and the caffeine/carbamazepine ratio.

Sample	Mass Fraction (ng g^−1^ d.w.)				CAF/CBZ
	Caffeine	Carbamazepine	Ciprofloxacin	Sulfamethoxazole	
LP 1	7.33	<LOQ	4.09	5.69	-
LP 2	10.98	<LOQ	5.33	12.68	-
LP 3	9.61	<LOQ	4.79	12.1	-
LP 4	29.70	1.6	28.08	38.85	7.22
LP 5	32.14	1.81	26.21	31.13	6.91
LP 6	24.97	1.61	22.44	40.02	6.03
LP 7	19.31	1.46	23.31	36.93	5.14
LP 8	22.33	1.13	19.97	32.96	7.68
LP 9	30.38	1.55	28.84	42.95	7.62
LP 10	21.31	1.7	25.21	40.7	4.87
LP 11	20.60	1.56	25.7	30.79	5.14
LP 12	19.53	1.49	25.51	36.13	5.10
LP 13	17.62	1.63	26.06	41.79	4.20
LP 14	16.02	<LOQ	23.57	33.28	-
LP 15	18.80	<LOQ	26.52	39.95	-
LP 16	27.12	1.54	27.01	36.82	6.89
LP 17	34.83	1.86	28.29	43.71	7.38
LP 18	37.68	1.61	31.04	44.77	9.12

<LOQ: below the limit of quantification (for carbamazepine LOQ = 1 ng g^−1^).

**Table 4:** Mass fraction levels of the target PhACs and total organic carbon in sediment from a core taken from the lowly impacted area in La Paz lagoon.

Depth (cm)	Mass Fraction (ng g^−1^ d.w.)				TOC (%)
	Caffeine	Carbamazepine	Ciprofloxacin	Sulfamethoxazole	
<5	13.17	4.14	7.51	14.56	0.68
6	13.11	3.72	8.17	14.69	0.34
9	10.30	2.89	5.83	8.82	0.22
15	9.18	1.21	4.59	7.17	0.15
17	7.83	<LOQ	3.04	6.14	0.08
18	5.36	<LOD	4.22	5.14	0.05
20	6.75	<LOD	2.73	3.78	0.05
21	7.16	<LOD	<LOQ	2.86	0.01
24	6.76	<LOD	<LOD	2.11	0.01
25	3.32	<LOD	<LOD	2.25	0.03

<LOD: below the limit of detection, <LOQ: below the limit of quantification (for carbamazepine LOD = 0.25 ng g^−1^ and LOQ = 1 ng g^−1^; for ciprofloxacin LOD = 0.5 ng g^−1^ and LOQ = 2 ng g^−1^).

**Table 5:** Calculated risk quotients for the target PhACs in superficial sediments. When *RQ* is <0.1 (values marked in green) the risk is extremely low, for *RQ* values between 0.01 and 0.1 (values marked in yellow) the risk is low, and for *RQ* surpassing 1 (values marked in red) the risk is classified as high.

Site	Risk Quotient Values			
	Caffeine	Carbamazepine	Ciprofloxacin	Sulfamethoxazole
LP 1	<0.01	0.01	0.01	1.18
LP 2	<0.01	0.01	0.01	2.40
LP 3	<0.01	0.01	0.01	2.29
LP 4	0.01	0.03	0.06	7.34
LP 5	0.01	0.04	0.05	5.88
LP 6	0.01	0.03	0.04	7.57
LP 7	<0.01	0.03	0.05	6.98
LP 8	<0.01	0.02	0.04	6.23
LP 9	0.01	0.03	0.06	8.12
LP 10	0.01	0.03	0.05	7.69
LP 11	0.01	0.03	0.05	5.82
LP 12	<0.01	0.03	0.05	6.83
LP 13	<0.01	0.03	0.05	7.90
LP 14	<0.01	0.02	0.05	6.29
LP 15	0.01	0.02	0.05	7.55
LP 16	0.01	0.03	0.05	6.96
LP 17	0.01	0.04	0.06	8.26
LP 18	0.01	0.03	0.06	8.46

## Data Availability

The raw data supporting the conclusions of this article will be made available by the authors upon request (The data is not available at this point because is used for a doctoral thesis).

## Supplementary Materials

The following supporting information can be downloaded at: https://www.mdpi.com/article/10.3390/jox14040093/s1, Table S1: Physical and chemical properties of the target PhACs; Table S2: Validation parameters of the analytical method to quantify the analytes in sediment samples; Table S3: Determination of predicted no-effect concentration (PNEC) of the target PhACs for aquatic species from different trophic levels using different endpoints in chronic toxicity assays; Table S4 [37,65,66,67]: Predicted no-effect concentrations of the target PhACs for different trophic levels in the aquatic environment and their adaptation for sediments; Figure S1: Matrix-matched calibration curves of the analytes: (a) carbamazepine, (b) sulfamethoxazole, (c) ciprofloxacin, and (d) caffeine.
