# Supplementary material for: Pharmaceutical Residues in Sediments of a Coastal Lagoon in Northwest Mexico—Occurrence and Environmental Risk Assessment
Source: J Xenobiot. 2024 Nov 7;14(4):1757–70. doi: 10.3390/jox14040093 (PMC11587066; doi:10.3390/jox14040093)
Supplement: Supplementary file 1 [file jox-14-00093-s001.zip › jox-3246468-supplementary.pdf]

Supplementary Information

Pharmaceutical Residues in Sediments of a Coastal Lagoon in Northwest Mexico—Occurrence and Environmental Risk Assessment

Oscar Fernando Becerra-Rueda, Griselda Margarita Rodríguez-Figueroa, Ana Judith Marmolejo-Rodríguez, Sergio Aguíñiga-García and Juan Carlos Durán-Álvarez

Table S1. Physical and chemical properties of the target PhACs

Compound	Structure	pK_a	log K_{ow}	Water solubility ($g\ L^{-1}$)	log D at pH = 8.4[a]	Henry's Law constant ($atm\ m^3\ mol^{-1}$)
Caffeine	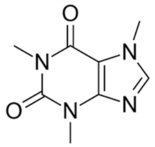	14	-1.32	21.7	-1.32	1.1×10^{-11}
Ciprofloxacin	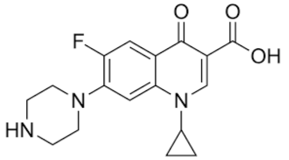	$pK_{a1} = 6.09$<br>$pK_{a2} = 8.62$	0.28	25	0.044	5.09×10^{-19}
Sulfamethoxazole	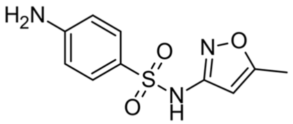	$pK_{a1} = 1.85$<br>$pK_{a2} = 5.6$	0.89	0.61	1.92	6.4×10^{-13}
Carbamazepine	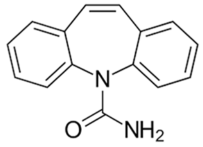	14	2.77	0.018	3.03	1.08×10^{-7}

[a] For calculations of the logD values, a pH = 8.4 was used as it is the average pH value of marine water in the site where sediment samples were collected

Table S2. Validation parameters of the analytical method to quantify the analytes in sediment samples

Parameter	n	Caffeine	Ciprofloxacin	Sulfamethoxazole	Carbamazepine
Recovery (%)					
30 ng g^{-1}	3	87.7-95.1	98-103	102-106	94.6-100
75 ng g^{-1}	3	86.2-91.4	95-101	97.5-104	96.9-102
125 ng g^{-1}	3	79.3-90.8	92-99.3	99.3-102	95.9-104
Precision (%, RSD)					
50 ng g^{-1}	5	3.5-4.4	3.7-4.9	3.6-4.4	2.5-2.8
100 ng g^{-1}	5	1.9-3.2	2.4-3.2	2.0-2.7	1.8-2.9
LOD (ng g^{-1})	-	0.25	0.5	0.5	0.25
LOQ (ng g^{-1})	-	1	2	2	1

Table S3. Determination of predicted no-effect concentration (PNEC) of the target PhACs for aquatic species from different trophic levels using different endpoints in chronic toxicity assays (Beiras, 2021).

Compound	Species	Endpoint	Toxicity threshold	AF	PNEC (ng L^{-1})
Caffeine	*I. galbana*	Population growth (3 d)	EC_{10}	100	192000
	D. magna	**Reproduction (21 d)**	**NOEC**	**100**	**1200**
	P. promelas	Mortality (7 d	EC_{50}	100	183330
Carbamazepine	*R. subcapitata*	Population growth (3 d)	EC_{10}	100	6000
	C. dubia	Reproduction (7 d)	LOEC	100	1000
	D. rerio	**Reproduction 42 d)**	**LOEC**	**100**	**5**
Ciprofloxacin	***M. aeruginosa***	**Population growth (7 d)**	**EC_{50}**	**100**	**17**
	D. magna	Neonate immobilization (2 d)	NOEC	100	600000
	P. promelas	Survival and growth (7 d)	EC_{50}	100	33330
Sulfamethoxazole	***C. neogracile***	**Cell density (20 d)**	**LOEC**	**100**	**0.75**
	C. dubia	Reproduction (7 d)	EC_{50}	100	700
	O. latipes	Mortality (4 d)	LC_{50}	1000	>333330

The PNEC values in bold were used to determine the risk quotients under the approach of the worst-case scenario

Table S4. Predicted no effect concentrations of the target PhACs for different trophic levels in the aquatic environment and their adaptation for sediments

Compound	$PNEC_{water}$ (ng L^{-1})	K_d (L kg^{-1})	$PNEC_{sediment}$ (ng g^{-1})
Caffeine	1200	4.9[a]	4,667
Carbamazepine	5	12.6[b]	50
Sulfamethoxazole	0.75	8.9[c]	5.29
Ciprofloxacin	17	37.05[d]	500

[a](da Costa Filho et al., 2022), [b](Azuma et al., 2017), [c](Xu et al., 2009), [d](Gibs et al., 2013)

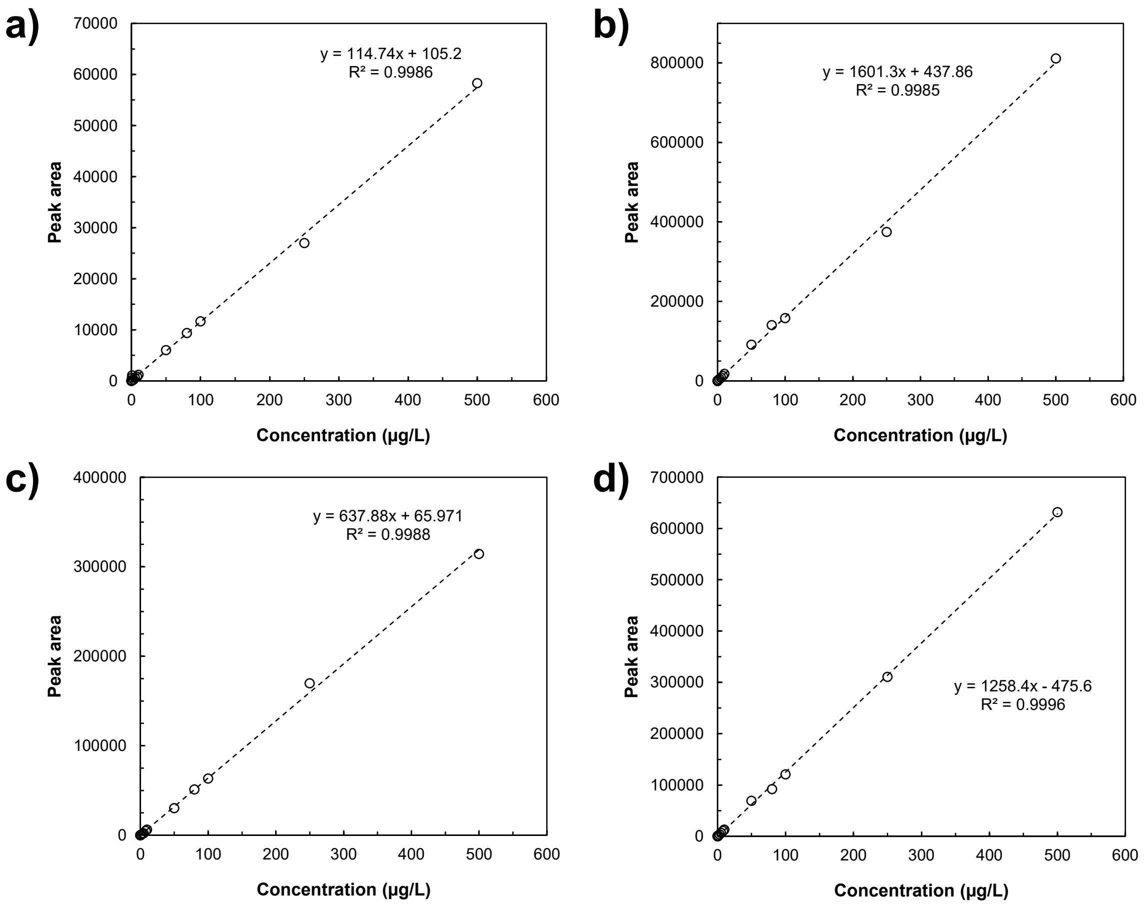

Figure S1. Matrix-matched calibration curves of the analytes: a) carbamazepine, b) sulfamethoxazole, c) ciprofloxacin, and d) caffeine

References

Azuma, T., Arima, N., Tsukada, A., Hirami, S., Matsuoka, R., Moriwake, R., Ishiuchi, H., Inoyama, T., Teranishi, Y., Yamaoka, M., Ishida, M., Hisamatsu, K., Yunoki, A., Mino, Y., 2017. Distribution of six anticancer drugs and a variety of other pharmaceuticals, and their sorption onto sediments, in an urban Japanese river. Environ. Sci. Pollut. Res. 24, 19021–19030. https://doi.org/10.1007/s11356-017-9525-0

da Costa Filho, B.M., Duarte, A.C., Santos, T.A.P.R., 2022. Environmental monitoring approaches for the detection of organic contaminants in marine environments: A critical review. Trends Environ. Anal. Chem. 33, e00154. https://doi.org/10.1016/j.teac.2022.e00154

Gibs, J., Heckathorn, H.A., Meyer, M.T., Klapinski, F.R., Alebus, M., Lippincott, R.L., 2013. Occurrence and partitioning of antibiotic compounds found in the water column and bottom sediments from a stream receiving two wastewater treatment plant effluents in Northern New Jersey, 2008. Sci. Total Environ. 458–460, 107–116. https://doi.org/10.1016/j.scitotenv.2013.03.076

Xu, W.H., Zhang, G., Wai, O.W.H., Zou, S.C., Li, X.D., 2009. Transport and adsorption of antibiotics by marine sediments in a dynamic environment. J. Soils Sediments 9, 364–373. https://doi.org/10.1007/s11368-009-0091-z
